# Nonadherence to psoriasis medication as an outcome of limited coping resources and conflicting goals: findings from a qualitative interview study with people with psoriasis

**DOI:** 10.1111/bjd.15086

**Published:** 2016-12-17

**Authors:** R.J. Thorneloe, C. Bundy, C.E.M. Griffiths, D.M. Ashcroft, L. Cordingley

**Affiliations:** ^1^ Centre for Dermatology ResearchManchester Academic Health Science CentreUniversity of ManchesterManchesterU.K.; ^2^ School of Biological SciencesManchester Academic Health Science CentreUniversity of ManchesterManchesterU.K.; ^3^ Centre for Pharmacoepidemiology and Drug SafetySchool of Health SciencesManchester Academic Health Science CentreUniversity of ManchesterManchesterU.K.; ^4^ Salford Royal NHS Foundation TrustManchesterU.K.; ^5^ Division of Musculoskeletal and Dermatological SciencesUniversity of ManchesterManchesterU.K.

## Abstract

**Background:**

Medication nonadherence is known to limit the effectiveness of available therapies; however, little is known specifically about medication adherence in people with psoriasis. Medicines self‐management can feel onerous to those with dermatological conditions due to the nature of therapies prescribed and many individuals with psoriasis experience additional challenges such as physical and psychological comorbidities that place significant additional demands on individuals and may undermine adherence. Viewing nonadherence to medication as an outcome of limited personal coping resources and conflicting goals may help to explain medication nonadherence.

**Objectives:**

To explore individuals’ perspectives of their psoriasis, medication and its management.

**Methods:**

Twenty people with psoriasis were recruited from community samples in England and interviewed in‐depth about their perceptions of their psoriasis, medication, and adherence to medication and self‐management advice. Data were analysed using Framework Analysis.

**Results:**

Participants reported that adhering to recommended treatment regimens conflicted with the management of the physical and psychological demands of living with psoriasis. Medication usage was viewed as a source of unresolved emotional distress and, for some, resulted in poor self‐reported adherence, which included medication overuse, underuse and rejection of prescribed therapies. Perceived lack of engagement by clinicians with participants’ self‐management difficulties was viewed as an additional source of stress and distress.

**Conclusions:**

Adhering to medication in psoriasis can be an additional source of considerable emotional distress. We interpreted some episodes of nonadherence to psoriasis medication as rational attempts by individuals to minimize distress and to gain control over their life.

## Abstract 2

**What's already known about this topic?**

- Although common, little is known about factors contributing to nonadherence to therapies used in psoriasis.

**What does this study add?**

- Illness and medication beliefs and associated mood influence medication nonadherence.
- Attempts to adhere can undermine people's ability to manage their emotional distress.
- For some patients, following prescribed medication regimens adds to rather than relieves psoriasis‐associated distress.

**What are the clinical implications of this work?**

- Some occurrences of nonadherence to medication in psoriasis should be recognized as a strategic decision by the patient to reduce distress and to gain control.
- Assessing patients’ beliefs and mood, and identifying potentially conflicting health goals may help to support medication adherence.

Psoriasis is a chronic immune‐mediated inflammatory skin condition, affecting at least 2% of the U.K. population.^1^ Individuals are often diagnosed before the age of 30 years^2^ and therefore will use pharmacological therapies for most of adulthood. Medication adherence, defined as the ‘extent to which the patients’ behaviour matches *agreed* recommendations from the prescriber’, is a major barrier to optimizing the efficacy of available therapies,^3^ with people typically taking only half of their prescribed medication.^4^ People with psoriasis express widespread dissatisfaction with medication,^5^, ^6^ view the provision of formal support for medicines use as poor^7^ and experience adherence difficulties,^8^, ^9^ yet few high‐quality studies have examined the influences on adherence to therapies used in psoriasis.^10^

Self‐regulation can be used to describe how individuals alter their thoughts or behaviours in order to achieve a desired goal.^11^ Leventhal's Common‐Sense Self‐Regulatory Model (CS‐SRM)^12^ can offer a useful framework for exploring how individuals respond to and cope with the emotional and behavioural demands involved in living with a long‐term condition (LTC).^13^ The framework describes the role of individual's illness *beliefs* in influencing psychological distress and the ability to effectively manage the condition.^7^, ^14^, ^15^, ^16^, ^17^

An extension of the CS‐SRM known as the Necessity‐Concerns Framework^18^ identifies medication‐specific beliefs as also playing a key role. Individual's beliefs about their personal need for medication to manage current and future health (specific‐necessity) are *balanced* against their concerns about potential negative effects from medication (specific‐concerns).^19^, ^20^ To date, no study has applied these psychological frameworks for understanding medication adherence in psoriasis.^10^

Medication adherence can be difficult, especially in situations where an individual must focus on their long‐term goals while forgoing short‐term gains and overcoming barriers, temptations or habits.^21^ For example, an individual may choose not to use their prescribed medication regularly if they are worried about adverse effects; this behaviour may temporarily improve mood, but they are no longer optimally managing their condition. In a synthesis of qualitative research on individuals’ perceptions of medication‐taking across various LTCs, Pound *et al*.^22^ found that appropriate medication usage may conflict with other priorities. These included social, work and study commitments, medication concerns and fear of stigmatization and disclosure, with these challenges influencing medication nonadherence. One additional challenge for those with psoriasis is the need to manage additional illnesses, both physical and psychological,^23^, ^24^, ^25^, ^26^, ^27^, ^28^ and the associated behavioural risk factors for example, management of weight reduction and specific dietary demands, smoking and excessive alcohol consumption,^29^, ^30^, ^31^ which can increase the risks of exacerbating psoriasis and of developing comorbidities.^32^ These physical and psychological demands of managing psoriasis place significant demands on the individual, and may increase the likelihood of adherence difficulties. In‐depth qualitative explorations of patient perspectives on medication use are rare in psoriasis research.^33^

Nonadherence to medication may be the result of limited personal resources, conflicting goals and priorities, and, in the psychological literature is termed self‐regulation failure.^21^, ^34^ Figure 1 depicts the key processes involved in a decisional analysis: (i) appropriate medication usage may result in the reduction of personal resources (e.g. time and motivation) to manage other tasks and (ii) appropriate medication usage may result in ongoing internal conflict between a desire to adhere and the need to address other priorities. The prevalence of individuals with strong beliefs in the necessity of medication *and* strong concerns about its usage,^35^ and continued psychological distress despite the successful reduction of symptoms in inflammatory conditions^36^ led us to undertake an in‐depth study of patients’ experiences of psoriasis medication usage.

**Figure 1:**
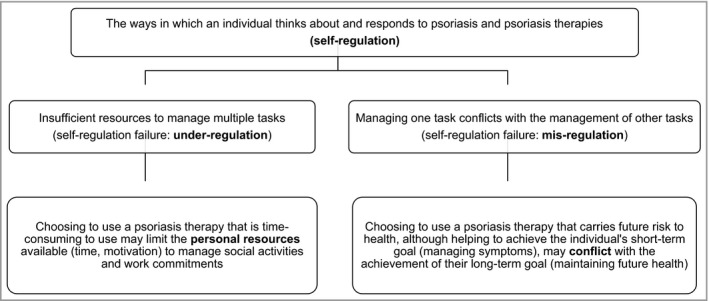
The challenges of medication adherence, in the context of self‐regulation failure.

The aim of this qualitative study was to explore individuals’ perspectives of their psoriasis, medication and its management.

## Methods

### Sampling and recruitment

Invitation e‐mails were sent to potential participants with a diagnosis of psoriasis recruited from community venues (places of worship, libraries, community halls) and a national established patient support association in the U.K. A purposive sampling strategy was used to obtain a diverse demographic and clinical group. Of 75 invitations sent to potential participants, 36 people demonstrated interest and were sent further details, with a final sample of 20 people recruited from the support website (*n* = 14) and from the community (*n* = 6) agreeing to participate in the study (Table 1).

**Table 1:** Demographic and disease characteristics of participants (*N* = 20)

Characteristic	*n*
Sex	
Female	9
Male	11
Ethnicity	
White	18
Indian	1
Chinese	1
Employment	
In paid work (full or part‐time, including self‐employed)	12
In full‐time education/training	3
Unemployed	1
Retired	3
Retired and voluntary work	1
Education	
1 or more ‘O’ level equivalents	1
1 or more ‘A’ level equivalents	2
Trade qualifications	3
Professional qualifications	8
Degree	6
Housing	
Owner‐occupied/mortgaged	14
Rented from local authority/housing association	2
Rented from a private landlord	3
Other (living with parents)	1
Psoriasis type	
Chronic plaque	19
Palmoplantar psoriasis	1
Comorbid conditions	
Psoriatic arthritis	3
Current treatments^a^	
Topical	16
Phototherapy/photochemotherapy	1
Oral systemic	6
Biologic	1
Nonprescription (e.g. tanning booths, Chinese medicine)	3
Past treatments^a^	
Topical	18
Phototherapy/photochemotherapy	12
Traditional systemic	7
Age, mean years (range)	
At study intake	47·2 (21–71)
When psoriasis started	23·4 (4–52)

a More than one characteristic can be recorded by each individual.

### Data collection

During October 2011 and February 2012, data were collected via face‐to‐face, in‐depth cognitive interviews with participants at a location convenient for them (Table S1, see Supporting Information). Interview questions about medication use were based on the self‐reported adherence items from the Medication Adherence Report Scale (MARS),^19^ and questions about medication beliefs were based on items from the Beliefs about Medicines Questionnaire (BMQ).^37^ Using a cognitive interviewing approach,^38^, ^39^ participants responded to items from the MARS and the BMQ while simultaneously providing in‐depth and detailed reasons for their responses. Additional questions were added by the interviewer to expand on individual's perceptions of psoriasis,^12^, ^40^ emotional distress and its management.

Individuals were recruited and interviewed sequentially with analysis being undertaken after each three to four interviews, and additional questions added to the interview guide if new relevant themes were identified. Data saturation (no new themes emerging) was achieved after 20 interviews, constituting the final sample for this study. Mean duration of the interviews was 90 min. They were conducted, audiorecorded and transcribed verbatim by R.J.T. Informed consent was obtained from all participants prior to data collection. Research Ethics Committee approval was obtained (reference 11059).

### Data analysis

Data were analysed based on the principles of Framework Analysis.^41^ Each interview was read repeatedly for the researcher to become familiar with the content and identify key issues and emergent themes (Stage 1). An a priori coding (thematic) framework was developed using the constructs of the CS‐SRM and the Necessity‐Concerns Framework (Stage 2). This framework was added to by combining the concepts, themes and relationships that emerged during the familiarization stage. The themes identified in Stage 1 were added as subthemes to the primary themes identified in the a priori framework template or included as additional primary themes. At this stage, reports of limited personal resources and goal conflict were identified as potentially relevant. All primary themes and subthemes were numbered to create a coding framework, which was then applied to all the transcripts systematically (Stage 3). Data were then extracted from the transcripts and sorted by primary theme and subtheme in a standard spreadsheet (Stage 4). During these latter stages, thematic associations and patterns were recorded (Stage 5). Informed by the original research aims, all themes were constantly compared within and across cases, in order to identify similar or contrasting themes, to identify disparities and to explore patterns and connections between themes.

One researcher (R.J.T.) undertook the preliminary analysis with the interpretative component undertaken using secondary validation by a second author (L.C.). The multidisciplinary research team comprising researchers from diverse professional backgrounds including psychology, dermatology and pharmacy, engaged in ongoing discussion to improve the transferability and credibility of the findings. The quality and transparency of this research was guided by the Standards for Reporting Qualitative Research (SRQR) recommendations.^42^

## Results

The analysis produced four overarching themes and subthemes (Table 2).

**Table 2:** Self‐regulation failure themes and subthemes

Theme	Description	Subtheme
Theme 1	Depletion of personal coping resources	1.1 Social activities
		1.2 Exercise and physical activity
		1.3 Work commitments
Theme 2	Conflict with the management of other illness tasks	2.1 Treatment concerns
		2.2 Uncertain treatment efficacy
		2.3 Stress as a causal factor of psoriasis
		2.4 Adjustment to psoriasis
Theme 3	Adherence to medication	3.1 Use medication in accordance with current guidelines
		3.2 Treatment holidays/breaks
		3.3 Reducing the frequency of topical application
		3.4 Increasing the duration of topical application
		3.5 Increasing topical therapy dosage
		3.6 Reject prescribed topical therapy
Theme 4	Relationships with healthcare professionals as an additional stressor	4.1 Lack of empathy with the challenges of medication usage
		4.2 Lack of support reinforced rejection of psoriasis therapies

### Theme 1: depletion of personal coping resources

Adherence to psoriasis therapies depleted personal coping resources, including time and motivation available for other illness tasks (Table 3). Participants reported the frequent collection of repeat topical prescriptions and high‐frequency applications as time‐consuming and requiring significant effort, which limited the time and motivation available to engage in social activities. Perceived stigmatization resulted in social avoidance, with individuals hiding their condition from others and actively avoiding public places or social situations. Topical therapy usage added to the use of these avoidant coping strategies; the deleterious effect on clothing (e.g. staining) and perceived stigmatization resulted in participants avoiding social situations when using topical therapies. Some believed they had to plan their use of topical therapies in advance of any social engagements, in order to ensure that its usage was not visible to others. Some participants found it difficult to participate in exercise activity due to the perceived stigma of psoriasis, and topical therapy usage was an additional barrier by limiting the time available to participate in physical activity. Furthermore, adherence to therapy was viewed as adding to employment burden; the need for regular attendance at clinic appointments for phototherapy/photochemotherapy was viewed as time‐consuming and participants found it extremely difficult to balance clinic attendance with work commitments.

**Table 3:** Theme 1: depletion of personal coping resources

Subthemes	Data extracts
1.1 Social activities	‘I was once sat by the pool and I heard this woman say, “Look at that woman's feet,” and I felt absolutely *dreadful*.’ [P1: using topical therapy]
	‘So it [topical therapy] practically destroys any chance of having a *social life* or anything like that.’ [P5: prescribed topical therapy but using tanning beds]
	‘… I'll *plan* it [topical therapy] if I know we will be going out or if we are going out for a family meal or whatever. I'll plan to use stuff so when we do go out, it's *not noticeable* that I've used anything.’ [P15: using topical therapy and photochemotherapy]
1.2 Exercise and physical activity	‘I feel it's quite difficult to do activities like *swimming* or anything which exposes too much of my arms and legs really.’ [P4: using topical therapy]
	‘You're meant to use it [topical therapy] twice a day, which is not practical … it cuts into the time that I would like to go for a *jog* … ’ [P5: prescribed topical therapy but using tanning beds]
1.3 Work commitments	‘But actually, you know, if you've got a *job*, even if it's just a 9 to 5 job; to go to your UVB 3 times a week for a 12‐week period, that's quite a lot. Let alone if you travel a lot. It then becomes *unsustainable*.’ [P6: using topical therapy, previously used phototherapy]
	‘You just can't do it [follow the topical therapy regimen]. Especially *working* and things like that, you just can't do it.’ [P2: using topical therapy]

### Theme 2: conflict with the management of other illness tasks

Adherence to psoriasis therapies conflicted with the management of other illness tasks (Table 4). Participants expressed ongoing internal conflict between the need to manage and gain control over psoriasis symptoms by using prescribed therapies, and concerns that those therapies have the potential for adverse effects or risks of damage to future health. Participants commonly expressed major concerns about adverse effects such as potential liver damage from methotrexate or skin thinning from topical corticosteroids. The perception of psoriasis as a LTC requiring life‐long medication heightened these medication concerns. Participants expressed anxiety about prior as well as future medication use with some viewing their exposure to a range of different psoriasis therapies as resulting in long‐term and potentially damaging accumulation of medication adverse effects. Some participants reported managing the distress caused by these conflicting goals by being vigilant to potential adverse skin damage resulting from the use of topical corticosteroids and phototherapy/photochemotherapy. However, combined with participant uncertainty in correctly identifying and detecting potential skin changes, this served to perpetuate ongoing anxiety.

**Table 4:** Theme 2: conflict with the management of other illness tasks

Subthemes	Data extracts
2.1 Treatment concerns	‘ … I worry about the damage to my *liver* with methotrexate and also for my skin with the creams as well, because I know the steroids can *damage your skin* long term.’ [P13: using topical and oral therapy]
	‘I don't like the injections, it's kind of really painful and I get really *nervous* about it.’ [P3: using a biologic]
	‘ … It worries me the fact that I've been treating psoriasis for over 40 years now and so I'm worried about the *effect of those treatments* I have had over those years.’ [P16: using topical therapy]
	‘I do kind of look at my skin but I can't tell [if it's thinning or not]. *How would you tell?* Nobody ever notices, when I go to give blood, no one ever comments on it or anything like that.’ [P2: using topical therapy]
	‘Sometimes I *worry* because I don't want to become too dependent on medication, because this medication could be taken away from me at any time. It really depends on the way the NHS funding goes.’ [P3: using a biologic]
2.2 Uncertain treatment efficacy	‘ … Sometimes I feel that the treatments aren't stopping the psoriasis from *increasing* and sometimes I feel that the psoriasis is quite resistant and will increase, *despite using the treatment* … I'm not sure overall, whether they are stopping it from getting worse.’ [P4: using topical therapy]
	‘ … If this treatment that I am having now stops working … *I don't even want to think about it really* … because I know that at my age and given the other health problems that I have got … it's eliminated other tablet forms of treatments that um … I've had to stop because of the side‐effects.’ [P1: using topical therapy]
	‘What makes me feel uncertain about it is, you know, what other side‐effects it could pose … could it make my psoriasis worse if I ever stop taking it … I do *worry* whether if I ever stopped the medication, could something trigger it off to come back much, much worse than it was before. So that's a worry.’ [P3: using a biologic]
	‘I find it somewhat *distressing* and I have to make the decision, not knowing really what the outcome is. Not knowing if I stop using a particular cream whether I'm going to get more symptoms or if I get less.’ [P4: using topical therapy]
2.3 Stress as a casual factor of psoriasis	‘I was under a lot of *stress* so I did get lots of flare‐ups.’ [P10: using oral therapy]
	‘ … Being fairly active, not being able to do want I want to do, is very, very annoying and it's highly stressful and it's a *vicious circle*. So that brings on psoriasis even worse.’ [P5: prescribed topical therapy but using tanning beds]
	‘ … It depends on *how stressed I get at work* … I can be using my creams and doing my daily routine and it will still manage to creep back up to a level where I don't find acceptable.’ [P15: using topical therapy and photochemotherapy]
	‘ … I can either miss my UVB, and I know it's not great and I know I'm prescribed a course of it, or I can tell the people who I work for that I can't do this trip because I've got my psoriasis treatment … the latter would *cause me so much more stress than the former and make my psoriasis worse*, so you have to make decisions about things.’ [P6: using topical therapy, previously used phototherapy]
	I've only been on it 5 months, and I haven't really noticed a major difference but then I have had quite a lot of stress … I think the stress has *counteracted* the effectiveness of the drug.’ [P3: using a biologic]
2.4 Adjustment to psoriasis	‘ … It's more *upsetting* when it comes back. Because you think, this is it, I've found a cure but *there isn't a cure*.’ [P14: using oral therapy]
	‘I feel really *self‐conscious* because I've got this sort of gunk all over my head … if I put it on in the morning, *I can't go out* until the evening … ’ [P11: using topical therapy]
	‘ … It's the most awful thing in the world; it smells like you are creosoting a fence. So *I don't want to put my partner through that*.’ [P11: using topical therapy]
	‘I used to put greasy stuff in and you would be all sticky and then you would have to wash it all out and that took ages to wash out. I'm almost having *nightmares* just thinking about it.’ [P7: using topical and oral therapy]

Perceived poor control of symptoms and the unpredictable response to topical therapy was a source of internal conflict and distress and led participants to question the necessity of their therapy. Furthermore, some questioned whether topical therapy usage could *increase* psoriasis severity and resistance to medication, which served to perpetuate feelings of anxiety. The inability to use alternative psoriasis therapies due to existing contraindications (e.g. comorbid conditions, adverse medication effects) generated a sense of unwanted medication dependency and lack of long‐term psoriasis control that served to increase anxiety and negative expectations of the future course of psoriasis and its management.

Stress was viewed as a causal factor of psoriasis and exacerbating flares. In addition, the psychological and social *consequences* of psoriasis were perceived as stressful and contributing to symptom severity. Participants viewed this vicious psoriasis–stress cycle as particularly difficult, resulting in beliefs of limited or no control and feelings of frustration or hopelessness. Participants also expressed the belief that stress interacted with medication response; some believed stress could interfere with the efficacy of topical therapies, which maintained feelings of limited control and distress. In addition, psoriasis therapies and efforts to adhere to them were also viewed as inherently stressful because of their impact on daily life.

The experience of relapsing and remitting flares was a key factor in participants’ inability to adjust to their psoriasis. The experience of unpredictable flares, *despite* medication usage, perpetuated feelings of limited control and psychological distress. The unattractive effects of topical therapies were constant reminders of the visibility of their condition and its psychological and social consequences. Furthermore, the perceived visibility and noticeability of topical therapy usage conflicted with the strong desire to hide the condition from others, amplifying concerns about the negative reactions of others. Many participants felt they had to conceal their condition from the general public, *as well as* from their family, close friends and spouse/partner. However, while recognizing the importance of topical therapies, participants found usage of them challenged their need to conceal their condition; participants described topical therapies as a burden to themselves (e.g. messy to use, stains clothing), and also to their partners or close family members.

### Theme 3: adherence to medication

Adherence to psoriasis therapies depleted personal resources and conflicted with the management of other illness tasks. Despite these challenges and self‐reported distress, some participants reported continuing to use their psoriasis therapy as advised to help control disease and improve quality of life (Table 5). However, in order to reduce distress, manage uncertainty, gain control and increase the resources available for other tasks, some participants unilaterally adjusted their topical therapy use based on *perceived* symptom severity.

**Table 5:** Theme 3: adherence to medication

Subtheme	Data extracts
3.1 Use medication in accordance with current guidelines	‘I think you would be so *withdrawn* if you didn't have any of those treatments … I would find it extremely *difficult to cope*.’ [P15: using topical therapy and photochemotherapy]
	‘I've only ever stopped taking it once and that was on the *instructions* of the doctor because they suspected a possible adverse reaction.’ [P3: using a biologic]
	‘I use it regularly every day because that's how it has been *prescribed*.’ [P1: using topical therapy]
3.2 Treatment holidays/breaks	‘*Every now and again* I have a day when I say sod it, that's it, I can't do this 7 days a week, I'm only going to do this 6 days a week.’ [P16: using topical therapy]
	‘If I'm working from home for a week, then I might *miss* my scalp stuff for a couple of days, purely because I think no one is going to see it.’ [P11: using topical therapy]
3.3 Reducing the frequency of topical application	‘ … I have been concerned in the past about *skin thinning* … I just basically respond to what I need.’ [P2: using topical therapy]
	‘I sometimes go 10 months without using any … sometimes I don't feel that it is bad enough. And that can sometimes be because it is not bad enough, or that it's not affecting my lifestyle so I just choose that I don't want to put on my creams.’ [P6: using topical therapy, previously used phototherapy]
3.4 Increasing the duration of topical application	‘Often I alter the dose if I'm feeling that my psoriasis is particularly bad at the time or if I need to get my psoriasis to a manageable level … *Leave it on* for longer, that kind of thing.’ [P11: using topical therapy]
3.5 Increasing topical therapy dosage	‘ … I haven't discovered over these years whether there is any way of telling whether *putting more and more on will work*, it just seems to be how my body reacts after a certain period of time.’ [P4: using topical therapy]
	‘If I'm honest, using certain creams, there is probably the odd tendency to use *too much*, rather than too little.’ [P16: using topical therapy]
3.6 Reject prescribed topical therapy	‘There is no point me taking the strongest medication now, because the moment you stop taking it, *it comes back worse within a week*. So you go through a lot of *effort, discomfort and inconvenience* for 1 week's benefit? That's just plain rubbish … ’ [P5: prescribed topical therapy but using tanning beds]
	‘I just *gave up* because it wasn't getting any better and it wasn't working so I wasn't prepared to keep paying for the treatments which were doing nothing for me.’ [P9: prescribed topical therapy but using herbal therapies]

Managing the time‐consuming nature of topical therapies was difficult and for some, this ongoing depletion of personal resources (including time and effort) resulted in the decision to have ‘treatment holidays/breaks’. Some participants actively chose to underuse their topical therapy to avoid experiencing ongoing conflict. Thus, topical therapies were used only when their psoriasis flares were perceived to be severe and requiring immediate symptom management. This strategically nonadherent behaviour enabled individuals to regain a manageable balance of addressing psoriasis symptoms and medication concerns while diminishing the impact of topical therapies on daily life, feelings of uncertainty and distress. Some participants had sporadic topical therapy overuse (increasing the *duration* of topical application), interspersed with topical therapy underuse, which appeared to be intentionally compensatory for lack of regular therapy use. For those who were using their topical therapies regularly, topical therapy overuse (increasing topical *dosage*) was done in order to address uncertainty about topical therapy efficacy and gain control over troubling symptoms.

For some participants, heightened medication concerns, feelings of low controllability and dissatisfaction with the management of symptoms resulted in the decision to prioritize mood management and other illness tasks and use alternative therapies, which included the use of indoor ultraviolet radiation exposure in the form of tanning beds.

### Theme 4: relationships with healthcare professionals as an additional stressor

Participants experienced medication use to be challenging and distressing, but participants believed this was rarely acknowledged in clinic (Table 6). Participants reported that clinicians appeared to lack empathy with the effects of psoriasis therapies and that neither their medication concerns nor inability to control symptoms were acknowledged. This added to the internal conflict between medication usage and medication concerns and uncertainty, maintaining psychological distress. Perceived unacknowledged goal conflict and severe distress seemed to exacerbate self‐management difficulties, limiting opportunities for shared treatment decision making and isolating participants. Most of those interviewed indicated that they made medication decisions alone and this led to deviations from recommended regimens in terms of both medication underuse and overuse. Some participants withdrew from conventional healthcare support and sought alternative therapies.

**Table 6:** Theme 4: relationships with healthcare professionals as an additional stressor

Subthemes	Data extracts
4.1 Lack of empathy with the challenges of medication usage	‘ … They don't *understand* that you are wearing stuff on your head that makes you smell like you're painting fences … it's really *frustrating*, they [clinicians] need to look at the impact of these treatments, maybe then they could understand why people aren't sticking to them.’ [P11: using topical therapy]
	‘My [GP] said, “Well use the coal tar during the day,” and I said, “Well I can't because it's too messy … ,” and all she said was “Oh, well just wear gloves.” But it's *impossible* as I'm working with papers all the time.’ [P9: prescribed topical therapy but using herbal therapies]
	‘It did kind of put me under a bit of stress, it affected my condition hugely. I really don't understand why [I was unable to be prescribed my preferred biologic], I mean, I try not to think about it too much but at the time it did cause me a lot of *worry and upset* … there might have been a very good reason behind it but they didn't want to share it with me.’ [P3: prescribed a biologic]
	‘I had to really look [possible side‐effects] up myself on the Internet. She [clinician] was quite blasé about it … and I was reading all these blogs about side‐effects which worried me, so I *wasn't happy* that I wasn't getting all the information.’ [P13: using topical and oral therapy]
	‘Nobody has ever really asked me how I feel about my psoriasis or how I deal with my psoriasis. Or what my feelings are. Nobody has ever *asked* me that.’ [P15: using topical therapy and photochemotherapy]
4.2 Lack of support reinforced rejection of psoriasis therapies	‘I stopped going [to the GP] because they weren't *helping* … because nothing [treatment] had worked there wasn't really much point in trying another one.’ [P9: prescribed topical therapy but using herbal therapies]

## Discussion

We undertook an in‐depth study of patient perspectives in treatment decision making and adherence to therapy in psoriasis. We found that individuals’ reported beliefs about their psoriasis and medication and associated mood played a crucial role in medication adherence. One notable finding is that medication underuse and overuse were influenced by distinct beliefs about psoriasis and psoriasis medication. Medication underuse seemed to be driven by concerns about the potential for adverse effects, perceived poor control of symptoms and feelings of anxiety. Medication overuse was influenced by experiences of unpredictable flares during medication usage, perceptions of uncertain treatment effectiveness and frustration.

These results suggest nonadherence to psoriasis medication may be a function of depleted personal resources and of conflicting demands or priorities, differing aspects of what is known as self‐regulation failure.^21^, ^34^ Topical therapy was viewed as time‐consuming and messy, and its use was viewed as increasing the likelihood of socially avoidant behaviours, creating additional employment/financial burdens and reducing or ending participation in physical activities. These factors are recognized problems in this population^43^, ^44^, ^45^ and all were perceived as contributing to ongoing psychological distress.

The need to manage psoriasis symptoms with prescribed medication conflicted with the need to manage concerns about adverse effects. Perceived uncertainty about treatment effectiveness and views that they had little control over symptoms added to internal conflict. Participants reported stress as a cause and trigger of psoriasis, and described ongoing struggles between their need to limit exposure to stressors and their desire to use psoriasis therapies perceived as stressful. Furthermore, the use of visible topical therapies undermined their desire to hide their condition from others. These challenges resulted in unresolved emotional distress, little perceived control and high levels of uncertainty. Findings reflected other evidence that highlighted perceived lack of support from healthcare providers, especially within general practice management,^7^, ^33^, ^46^ and in the current study this further undermined optimal medication usage.

Despite these challenges, some people reported continuing to use their psoriasis therapy as prescribed, although this led to continued high levels of unresolved distress. Others managed this distress by adjusting their medication usage based on perceived symptom severity, resulting in medication nonadherence, both medication underuse and overuse. A further group chose to reject their prescribed medication altogether and seek support from alternative sources.

Factors influencing medication adherence in psoriasis are largely unknown, with few studies exploring the role of patient perspectives in treatment decision making.^10^ Demographic and disease factors have inconsistent relationships with adherence, both in psoriasis^10^ and in other LTCs.^47^ Understanding the modifiable factors (patient perceptions) for nonadherence can help to develop effective adherence interventions. Increased distress and low patient satisfaction with care and therapy are associated with nonadherence in psoriasis^10^ and we found that individual's beliefs about psoriasis and its medication also play an important role in medication adherence. Complex adherence challenges and strategic modification of treatment regimens have been reported in other LTCs^22^ and this study demonstrates that similar challenges are also present in psoriasis. Nonadherence to medication may be viewed as an outcome of limited personal resources and conflicting goals, and therefore seen as a strategic and rational attempt to actively manage and limit the impact of ongoing emotional distress that results from conflicting demands and priorities.

The majority of participants recruited were using topical therapies and thus the findings may not be transferable to individuals whose treatment included more complex regimens. We acknowledge the potential for sampling bias; self‐reported distress and illness and medication burden in this study could be due to participants primarily being recruited through support groups. Additionally, levels of adherence disclosed by the participants were self‐reported rather than assessed by an objective measure. Despite these limitations, our qualitative methods have provided new insights and a richer understanding of factors that influence adherence in psoriasis and individuals’ perceptions of the challenges of treatment decision making, which have not been captured in previous survey studies. The SRQR guidelines^42^ informed the design, conduct and analysis of this qualitative research, resulting in high‐quality data and contributed to the credibility of the findings.

We suggest using a nonjudgmental approach that recognizes barriers to adherence^48^ and encouraging patients to discuss key factors that may influence nonadherence: their beliefs about psoriasis; medication and its management; how medication fits into their lives and any barriers they face. This approach may help patients identify potential solutions, help support psychological well‐being and enhance medication adherence. The identification and explicit recognition of potentially conflicting goals may itself be therapeutic and reduce distress.^49^ It is important to recognize that patients can still be experiencing considerable distress and illness and treatment uncertainty, even if they are adhering to their medication. Future research should use these findings to inform the development and evaluation of an adherence intervention and utilize a longitudinal study design to examine the prospective relationships between illness and medication beliefs and psychological distress with adherence to psoriasis medication.

## Supporting information


**Table S1.** Cognitive interview topic guide.

**Video S1**. Author video
