# Supplementary material for: Nonadherence to psoriasis medication as an outcome of limited coping resources and conflicting goals: findings from a qualitative interview study with people with psoriasis
Source: Br J Dermatol. 2016 Dec 17;176(3):667–76. doi: 10.1111/bjd.15086 (PMC5363250; doi:10.1111/bjd.15086)
Supplement: Supplementary file 1 — Table S1. Cognitive interview topic guide. [file BJD-176-667-s001.docx]

**Supporting information**

**Box S1** Cognitive interview topic guide

| - How do you use your prescribed therapy?^a^ |
| --- |
| - - Altering the dose, forgetting to use the medication, stopping medication for a while, missing a dose, taking less/more medication than instructed, avoiding using medication, using medication regularly, only using medication when experiencing a flare, using non-prescription therapies, clinic attendance |
| - - *Why is that? How does it make you feel (mood)?* |
| - To what extent do you view your prescribed therapy as necessary for managing your psoriasis?^b^ |
| - - Impact of medication on current and future physical symptoms and psychological and social well-being, treatment controllability |
| - - *Why is that? How does it make you feel (mood)?* |
| - To what extent do you have any concerns about your prescribed therapy or medicines in general?^b^ |
| - - Short- and long-term medication side-effects, current and medication disruptions to daily life, physical and psychological medication dependency, the overuse and harmfulness of medicines in general   - What do you understand about your prescribed therapies (how it works and how to use it)?   - *Why is that? How does it make you feel (mood)?* |
| Additional interview items to expand on participant’s responses^c^: |
| - Symptoms associated with psoriasis, impact of psoriasis on psychological and social well-being - What do you understand about your psoriasis (causes and triggers)? - *Why is that? How does it make you feel (mood)?*   - Relationships with health care professionals   - Shared treatment decision-making   - *Why is that? How does it make you feel (mood)?* |

*Key: a = items adapted from the Medication Adherence Report Scale^19^; b = items adapted from the Beliefs about Medicines Questionnaire^37^; c = items adapted from the Illness Perception Questionnaire- Revised^40^.*
